# Roles of hypoxic environment and M2 macrophage-derived extracellular vesicles on the progression of non-small cell lung cancer

**DOI:** 10.1186/s12890-023-02468-7

**Published:** 2023-07-03

**Authors:** Xiao Chu, Zetian Wang, Weiqing Wang, Wenjing Liu, Yunyun Cao, Liang Feng

**Affiliations:** 1 Department of Thoracic Surgery, The Fifth People’s Hospital of Shanghai, Fudan University, Shanghai, 200240 China; 2 Department of Trauma-Emergency & Critical Care Medicine, The Fifth People’s Hospital of Shanghai, Fudan University, Shanghai, 200240 China; 3 School of Medicine, The International Peace Maternity and Child Health Hospital, Shanghai Jiao Tong University, Shanghai, 200030 China; 4 Department of Surgical Oncology, Minhang Branch, Fudan University Shanghai Cancer Center, NO.106, Ruili Road, Minhang District, Shanghai, 200240 China

**Keywords:** Non-small cell lung cancer, Hypoxia, M2 macrophages, Extracellular vesicles

## Abstract

**Background:**

Hypoxia contributes to the development of invasive and metastatic cancer cells, and is detrimental to cancer treatment. This study aimed to explore the molecular mechanisms by which hypoxic microenvironments affect hypoxic non-small cell lung cancer (NSCLC) development and the effects of M2 macrophage-derived extracellular vesicles (EVs) on NSCLC cells.

**Methods:**

A549 cells were cultured in an anoxic incubator for 48 h to construct hypoxic A549 cells, and then normal and hypoxic A549 cells were harvested for RNA sequencing. Next, THP-1 cells were used to induce M2 macrophages, and EVs were isolated from THP-1 cells and M2 macrophages. Cell counting kit-8 and transwell assays were used to determine the viability and migration of hypoxic A549 cells, respectively.

**Results:**

After sequencing, 2426 DElncRNAs and 501 DEmiRNAs were identified in normal A549 cells and hypoxic A549 cells. These DElncRNAs and DEmiRNAs were significantly enriched in “Wnt signaling pathway,” “Hippo signaling pathway,” “Rap1 signaling pathway,” “calcium signaling pathway,” “mTOR signaling pathway,” and “TNF signaling pathway.” Subsequently, ceRNA networks consisting of 4 lncRNA NDRG1 transcripts, 16 miRNAs and 221 target mRNAs were built, and the genes in the ceRNA networks were significantly associated with “Hippo signaling pathway” and “HIF-1 signaling pathway.” EVs were successfully extracted from THP-1 cells and M2 macrophages, and M2 macrophage-derived EVs significantly enhanced the viability and migration of hypoxic A549 cells. Finally, M2 macrophage-derived EVs further upregulated the expression of NDRG1-009, NDRG1-006, VEGFA, and EGLN3, while downregulating miR-34c-5p, miR-346, and miR-205-5p in hypoxic A549 cells.

**Conclusions:**

M2 macrophage-derived EVs may worsen the progression of NSCLC in a hypoxic microenvironment by regulating the NDRG1-009-miR-34c-5p-VEGFA, NDRG1-006-miR-346-EGLN3, NDRG1-009-miR-205-5p-VEGFA, and Hippo/HIF-1 signaling pathways.

**Supplementary Information:**

The online version contains supplementary material available at 10.1186/s12890-023-02468-7.

## Background

Lung cancer is the primary cause of cancer-related deaths worldwide, accounting for 18.4% of all cancer deaths [1]. Lung cancer can be divided into two main tissue types: small cell cancer and non-small cell lung cancer (NSCLC). NSCLC accounts for 85% of all lung cancer cases [2]. The incidence of NSCLC is associated with smoking, environmental exposure, ionizing radiation, air pollution, chronic lung infections, and genetic factors. Currently, the widely used clinical treatments for NSCLC mainly include surgery, chemotherapy, radiotherapy, targeted therapy, and immunotherapy; however, the specific treatment depends on the stage of the disease and the patient’s health status. Few patients with early stage NSCLC require surgical treatment, and the 5-year survival rate of patients with stage IA NSCLC is 70% [3]. However, for patients with advanced NSCLC, surgery combined with chemotherapy or radiation therapy is necessary, and the overall 5-year survival rate is only 14–17%, with a poor prognosis [4]. The hypoxic microenvironment is a classic feature of many human malignancies that contributes to aggressive and metastatic tumor phenotypes and plays an essential role in tumor growth and development, angiogenesis, metastasis, and treatment resistance [5]. Therefore, it is important to further investigate the relevant mechanisms of NSCLC progression in a hypoxic microenvironment and to identify novel targets for the treatment of NSCLC.

Non-coding RNAs (ncRNAs) are mainly divided into two types according to their length, including microRNAs (miRNAs, approximately 20 nt) and long non-coding RNAs (lncRNAs, > 200 nt) [6, 7]. Recently, increasing evidence has shown that abnormal miRNA expression is closely associated with the occurrence, progression, recurrence, and metastasis of tumors [8, 9]. Various miRNAs are involved in the initiation and development of lung cancer; for example, miR-23a is upregulated in NSCLC cell lines, and miR-23a overexpression inhibits E-cadherin expression and stimulates epithelial-mesenchymal transition (EMT) in A549 cells [10]. Compared to adjacent normal tissues or normal cell lines, the levels of miR-9-5p, miR-1246, miR-330-3p, miR-93, and miR-128-3p are significantly increased in NSCLC tissues and cell lines [11]. In addition, lncRNAs play essential roles in many biological processes, including cell proliferation, apoptosis, invasion, metabolism, and metastasis, and have become a research hotspot in recent years [12]. LncRNAs, which serve as competitive endogenous RNAs (ceRNAs), can sponge miRNAs through miRNA response elements, thus participating in the occurrence and development of NSCLC [13]. Wang et al. [14] showed that the lncRNA SOX21-AS1 was highly expressed in lung cancer tissues and cells, and that SOX21-AS1 upregulated PIM2 expression by competitively binding to miR-24-3p, thereby promoting the proliferation, migration, and invasion of lung cancer cells. Another study demonstrated that lncRNA-XIST was abnormally overexpressed in NSCLC tissues and cell lines compared to their corresponding controls, and that the downregulation of XIST suppressed NSCLC progression by triggering cell pyroptosis mediated by the miR-335/SOD2/ROS signaling pathway [15]. Hypoxia can affect the progression, metastasis, and metabolism of NSCLC cells. In the hypoxic microenvironment of NSCLC, lncRNA AC020978 was up-regulated, and hypoxia-induced AC020978 could promote proliferation and glycolytic metabolism in NSCLC by regulating the PKM2/HIF-1α axis [16]. Additionally, FAM13A was reported to be induced by hypoxia in NSCLC, and silencing of FAM13A resulted in decreased proliferation and metastatic potential of NSCLC cells under hypoxic conditions [17]. These reports reveal the key roles of RNAs in the growth of NSCLC cells under normal and hypoxic conditions. However, the mechanisms by which hypoxia promotes the development of NSCLC remain unclear.

Extracellular vesicles (EVs) exist in almost all cells as carriers of DNA, RNA (mRNA, miRNA, and lncRNA), proteins, and lipids and are able to change the fate of recipient cells through autocrine and paracrine [18]. EVs are important tools for communication between cells and have attracted increasing attention. A previous study found that hepatocellular carcinoma (HCC) cell-associated EVs could be actively internalized by adipocytes, resulting in significant transcriptome changes; particularly, it was found that the induction of inflammatory phenotypes in adipocytes in addition to HCC cell-associated EVs could facilitate tumor growth, enhance angiogenesis, and recruit more macrophages by activating NF-κB pathway [19]. Another study demonstrated that NSCLC-derived EVs can promote the growth of NSCLC cells through the transmission of lncRNA UFC1 [20]. Hypoxia can alter the cargo and biological functions of cell-derived EVs and, compared to normal oxygen, the release of EVs increases after hypoxia [21]. Wang et al. [22] demonstrated that hypoxia exacerbated cisplatin resistance in NSCLC cells and hypoxia-induced EVs PKM2 transmitted cisplatin resistance to sensitive NSCLC cells in vitro and in vivo; thus, EVs PKM2 may be a promising therapeutic target for cisplatin resistance in NSCLC. Another report showed that intermittent hypoxia could enhances the function of EVs derived from lung cancer cells to aggravate the immunosuppressive state of macrophages [23]. These findings indicate that EVs have crucial effects on tumor development in hypoxic environments. Macrophages are differentiated cells of the mononuclear phagocyte lineage with special phenotypic characteristics and specific markers that promote tumor occurrence and progression. M2 macrophages, also known as surrogate-activated macrophages, have anti-inflammatory, wound-healing, tissue repair, and antigen presentation functions [24]. M2 macrophage-released EVs promote the metastasis and invasion of colorectal cancer cells [25]. However, the effects of M2 macrophage-derived EVs on hypoxic NSCLC require further exploration.

Therefore, this study first constructed hypoxic NSCLC cells and used RNA sequencing to explore the ceRNA mechanisms of the hypoxic microenvironment affecting NSCLC development. Next, THP-1 cells were used to induce M2 macrophages and EVs were isolated from THP-1 and M2 macrophages to investigate the effects of M2 macrophage-derived EVs on hypoxic NSCLC cells. Our study reveals the molecular mechanisms of NSCLC in a hypoxic microenvironment and provides new ideas for controlling NSCLC progression and treatment.

## Materials and methods

### Cell culture

The human NSCLC cell lines A549 and THP-1 were purchased from the Cell Bank of the Chinese Academy of Sciences, Shanghai (Shanghai, China). A549 cells were maintained in Dulbecco’s modified Eagle’s medium (DMEM; Thermo Fisher Scientific, Waltham, MA, USA) supplemented with 10% fetal bovine serum (FBS; Thermo Fisher Scientific). THP-1 cells were cultured in Roswell Park Memorial Institute 1640 medium (RPMI-1640; Thermo Fisher Scientific) containing 10% FBS. Under normal conditions, the two cell lines were cultured in an incubator with 5% carbon dioxide at 37 °C. For hypoxic A549 cells, A549 cells were incubated in an anoxic incubator with 1% oxygen concentration at 37 °C for 48 h.

### Whole transcriptome sequencing and bioinformatics analysis

The A549 cells under normal conditions (n = 3, blank group) and hypoxic A549 cells (n = 3, hypoxic group) were collected and sent to Yanzai Biotechnology (Shanghai) Co. Ltd. (Shanghai, China) for whole-transcriptome sequencing, including lncRNA and miRNA sequencing. Briefly, total RNA was isolated from each sample using the RNAiso Plus kit (Trizol, Takara Biomedical Technology Co., Ltd.) following the manufacturer’s recommendations. Then, the purity, concentration, and integrity of the total RNA were evaluated by NanoDrop™ One/OneC, Qubit™ RNA HS Assay Kit, and Agilent 4200 TapeStation system, respectively. After library construction of the lncRNAs and miRNAs, each sample was sequenced on an Illumina platform.

After data preprocessing and analysis, clean data were mapped to their corresponding databases using HISAT2 and Burrows-Wheeler Aligner software. Subsequently, differentially expressed miRNAs (DEmiRNAs) and differentially expressed lncRNAs (DElncRNAs) between normal A549 cells and hypoxic A549 cells were screened using the DESeq algorithm in the R package with the criteria of |log_2_Fold change (FC)| > 1 and adjusted p-value (padj) < 0.05. The identified DE miRNAs and DElncRNAs were subjected to functional analyses, including Gene Ontology (GO) terms and Kyoto Encyclopedia of Genes and Genomes (KEGG) pathway enrichment. Statistical significance was set at P < 0.05.

### Construction of ceRNA networks

According to the identified DElncRNAs, lncRNA NDRG1 (log_2_ FC = 6.94, padj = 3.52E-19) was found to be highly expressed in normal A549 cells and hypoxic A549 cells, and is closely associated with hypoxia [26]. Therefore, we selected lncRNA NDRG1 to establish ceRNA networks. A tool, miRanda (http://cbio.mskcc.org/miRNA2003/miranda.html), was used to forecast the lncRNA-miRNA connection pairs (Gap Extend = 0, Score Threshold = 80, Energy Threshold = -20, and matched seq% Threshold = 80%). Concurrently, connection pairs with opposite directions of lncRNA NDRG1 and the corresponding DE miRNAs were retained. The target genes of the aforementioned DEmiRNAs were predicted using the StarBase version 2.0 database (http://starbase.sysu.edu.cn/), and the connection pairs with opposite directions of DEmiRNAs and target genes were retained. The Pearson Correlation Coefficient between the target genes and lncRNA NDRG1 was calculated using the COR function (http://77.66.12.57/R-help/cor.test.html) in the R3.6.1 package and link pairs with *P* < 0.05. Finally, ceRNA networks of NDRG1 were constructed and visualized using Cytoscape (version 3.6.1; https://cytoscape.org/). Then, the lncRNAs, miRNAs, and target genes in the ceRNA networks were used for GO term and KEGG pathway enrichment analyses, and a padj value < 0.05 was selected as the screening threshold.

### Real-time quantitative PCR (RT-qPCR)

Based on the established ceRNA network, three ceRNA pairs (NDRG1-009-hsa-miR-34c-5p-VEGFA, NDRG1-006-hsa-miR-346-EGLN3, and NDRG1-009-hsa-miR-205-5p-VEGFA) were subjected to RT-qPCR analysis. Briefly, total RNA was reverse-transcribed into cDNA using the PrimeScript II 1st Strand cDNA Synthesis Kit (Takara Biomedical Technology Co., Ltd.) following the manufacturer’s instructions. The levels of related miRNAs were measured using the stem ring method, and U6 was used as the standard gene. To determine the expression levels of lncRNAs and mRNAs, glyceraldehyde-3-phosphate dehydrogenase (*GAPDH*) was used as the housekeeping gene. The sequences of all primers are listed in Table 1. The reaction of RT-qPCR was completed as follows: 95 °C for 10 min, a total of 40 cycles at 95 °C for 15 s and 60 °C for 60 s, 95 °C for 15 s, 60 °C for 60 s, and 95 °C for 15 s. The expression levels of lncRNA NDRG1, hsa-miR-34c-5p, hsa-miR-205-5p, hsa-miR-346, mRNA *VEGFA*, and mRNA *EGLN3* were calculated using the 2^−ΔΔCt^ method [27].

**Table 1:** Sequences of all primers for real-time quantitative PCR

Primer	Sequence (5’-3’)
hsa-miR-34c-5p-JH	GTCGTATCCAGTGCAGGGTCCGAGGTATTCGCACTGGATACGACGCAATC
hsa-miR-34c-5p-F	GCAGGCAGTGTAGTTAGCT
hsa-miR-205-5p-JH	GTCGTATCCAGTGCAGGGTCCGAGGTATTCGCACTGGATACGACCAGACT
hsa-miR-205-5p-F	GCTCCTTCATTCCACCGG
hsa-miR-346-JH	GTCGTATCCAGTGCAGGGTCCGAGGTATTCGCACTGGATACGACAGAGGC
hsa-miR-346-F	TGTCTGCCCGCATGCCT
Downstream universal primer	GTGCAGGGTCCGAGGT
U6-F	CTCGCTTCGGCAGCACA
U6-R	AACGCTTCACGAATTTGCGT
LncRNA NDRG1-009-hF	GTGATTGCGGCAGTTC
LncRNA NDRG1-009-hR	GTGCGGGACCGCATCAGGCGGGTCA
LncRNA NDRG1-006-hF	ACCGCCAGCACATTG
LncRNA NDRG1-006-hR	CACCACGGCATCCACT
VEGFA-hF	AGGGCAGAATCATCACGAAGT
VEGFA-hR	AGGGTCTCGATTGGATGGCA
EGLN3-hF	CTGGGCAAATACTACGTCAAGG
EGLN3-hR	GACCATCACCGTTGGGGTT
TGF-β-hF	GGCCAGATCCTGTCCAAGC
TGF-β-hR	GTGGGTTTCCACCATTAGCAC
IL-10-hF	GACTTTAAGGGTTACCTGGGTTG
IL-10-hR	TCACATGCGCCTTGATGTCTG
Arg-1-hF	GTGGAAACTTGCATGGACAAC
Arg-1 -hR	AATCCTGGCACATCGGGAATC
YM1-hF	ATCCAGTCTGGCTATGAGATCC
YM1-hR	TCAGTCGGGTATTTGTAGAGGG
CD206-hF	TCCGGGTGCTGTTCTCCTA
CD206-hR	CCAGTCTGTTTTTGATGGCACT
CD163-hF	GCGGGAGAGTGGAAGTGAAAG
CD163-hR	GTTACAAATCACAGAGACCGCT
GAPDH-hF	TGACAACTTTGGTATCGTGGAAGG
GAPDH-hR	AGGCAGGGATGATGTTCTGGAGAG

### Induction of M2 macrophages

THP-1 cells were used to induce M2 macrophages, as previously described [28]. Briefly, THP-1 cells were divided into the control and M2 macrophage groups. Cells in the control group were cultured in RPMI RPMI-1640 with 10% FBS. However, the cells in the M2 macrophages group were treated with 200 ng/mL phorbol myristate acetate (PMA) for 24 h. After washing three times with PBS, the cells were incubated in fresh RPMI-1640 medium supplemented with interleukin-4 (IL-4, 20 ng/mL) and IL-13 (20 ng/mL). After cultured for another 72 h, total RNA was extracted from the cells in the two groups, and the expression levels of M2 macrophages-related markers (TGF-β, IL-10, Arg-1, YM1, CD206, and CD163) were determined to assess the formation of M2 macrophages using RT-qPCR. The sequences of all primers are listed in Table 1.

### Isolation and characterization of EVs

The successfully induced cells (M2 macrophages) and THP-1 cells were both harvested for EVs isolation using ultracentrifuge at 4 °C. Briefly, the cell suspension was centrifuged at 500 ×g for 5 min, followed by 2000 ×g for 30 min, and 10,000 ×g for 60 min. Then, the supernatant was collected, and filtered using 0.22-µm filters (Millipore, USA). The supernatant obtained through filtration was transferred to an ultra-high-speed centrifuge tube and centrifuged at 120,000 ×g for 70 min. After removing the supernatant, the sediments were resuspended with 200 µL PBS, which was EVs.

The concentrations of isolated EVs were measured using a BCA protein assay kit (TAKARA). Transmission electron microscopy (TEM, JEOL LTD, MA, USA) was used to observe the morphology of the EVs, following the methods of Zhu et al. [29]. According to the protocols of Soares et al. [30], the particle size and distribution of the EVs were analyzed using a NanoSight NS300 particle size analyzer (NTA, Malvern Panalytical, Malvern, UK). Finally, western blotting was performed to detect the expression of EV-specific markers, including CD9, HSP70, and TSG101, using their corresponding antibodies (1:500, Proteintech Group, Inc., IL, USA), as described previously [31].

### Co-culture of EVs and hypoxic A549 cells

EVs derived from THP-1 cells and M2 macrophages were labeled with PKH67 (green fluorescence) using a PKH67 staining kit (Sigma-Aldrich, USA) according to the manufacturer’s instructions. Briefly, 700 µL EVs were mixed with 1300 µL Diluent C, and then 2 mL Diluent C containing with 16 µL PKH67 was added to the mixture. After 5 min of incubation at room temperature, 4 mL of 1% bovine serum albumin (BSA) was added to stop staining. After centrifuged at 12,000 ×g for 90 min at 4 °C, the sediments were resuspended with 300 µL PBS, which was PKH67-labeled EVs.

Hypoxic A549 cells were seeded in 24-well plates (5 × 10^4^ cells/well) and cultured overnight. The next day, 10 µL PKH67-labeled EVs were added to the cells and cultured for 24 h. After washing, the cells were fixed with 4% paraformaldehyde for 20 min and treated with 0.1% Triton-X for 20 min. After washing with PBS three times, 3% BSA was added and the cells were incubated for 1 h. After washing, mounting medium supplemented with DAPI was added, and images were acquired using a laser scanning confocal microscope (Leica Microsystems, Inc., USA).

### Cell viability and migration assays

To select the optimal concentration of EVs, different concentrations of EVs were used to treat hypoxic A549 cells, and cell viability was determined using the cell counting kit-8 (CCK-8, Shanghai Bioscience Technology Co. Ltd., Shanghai, China) according to the manufacturer’s protocols. Briefly, A549 cells were seeded into a 96-well plate at a density of 1 × 10^4^ cells/well and cultured in an anoxic incubator at 37 °C for 24 h. On the next day, the cells were treated with different concentrations (10 µg/mL, 20 µg/mL, and 50 µg/mL) of THP-1 cells-derived EVs and M2 macrophages-derived EVs. After incubated in an anoxic incubator for 24 h, 10 µL CCK-8 reagent was added to each well. After 0.5 h of incubation, a microplate reader (Thermo Fisher Scientific) was employed to examine the optical density at 450 nm.

The migration of hypoxic A549 cells with different treatments was assessed using Transwell chambers (pore size 8 μm; BD Biosciences, Beijing, China). A549 cells with different treatments were seeded into the upper Transwell chambers of a 24-well plate at a density of 1.5 × 10^5^ cells/well; 500 µL of complete medium was in the lower chambers. After culturing for 24 h, the transwell chambers were removed, washed with PBS, and fixed with 4% paraformaldehyde at room temperature for 10 min. After two washes with PBS, the cells were stained with 0.5% crystal violet for 10 min. After removing excess dye and washing, the cells were observed under a microscope and photographed.

### Statistical analysis

Each experiment was repeated three times, and data were reported as mean ± standard deviation (SD). GraphPad Prism 5 (GraphPad Software, San Diego, CA, USA) was used for all the statistical analyses. Student’s *t*-test was used to compare the two groups. For the comparison of more than two groups, a one-way analysis of variance (ANOVA) followed by Tukey’s test was used. A *P* value less than  0.05 was considered to be statistically significant.

## Results

### Hypoxia accelerated the growth of A549 cells

CCK-8 was used to determine the viability of A549 cells under normal and hypoxic conditions. It was found that the cell viability in the A549 and hy-A549 cells was 100 ± 10.96% and 125 ± 15.72%, respectively (**Figure**S1). Hypoxic conditions significantly enhanced the viability of A549 cells (*P* < 0.05, **Figure**S1), indicating that A549 cells under hypoxic conditions may grow faster than their counterparts under normal conditions.

### Identification of DElncRNAs between normal and hypoxic A549 cells and functional analysis of these DElncRNAs

**Fig. 1:**
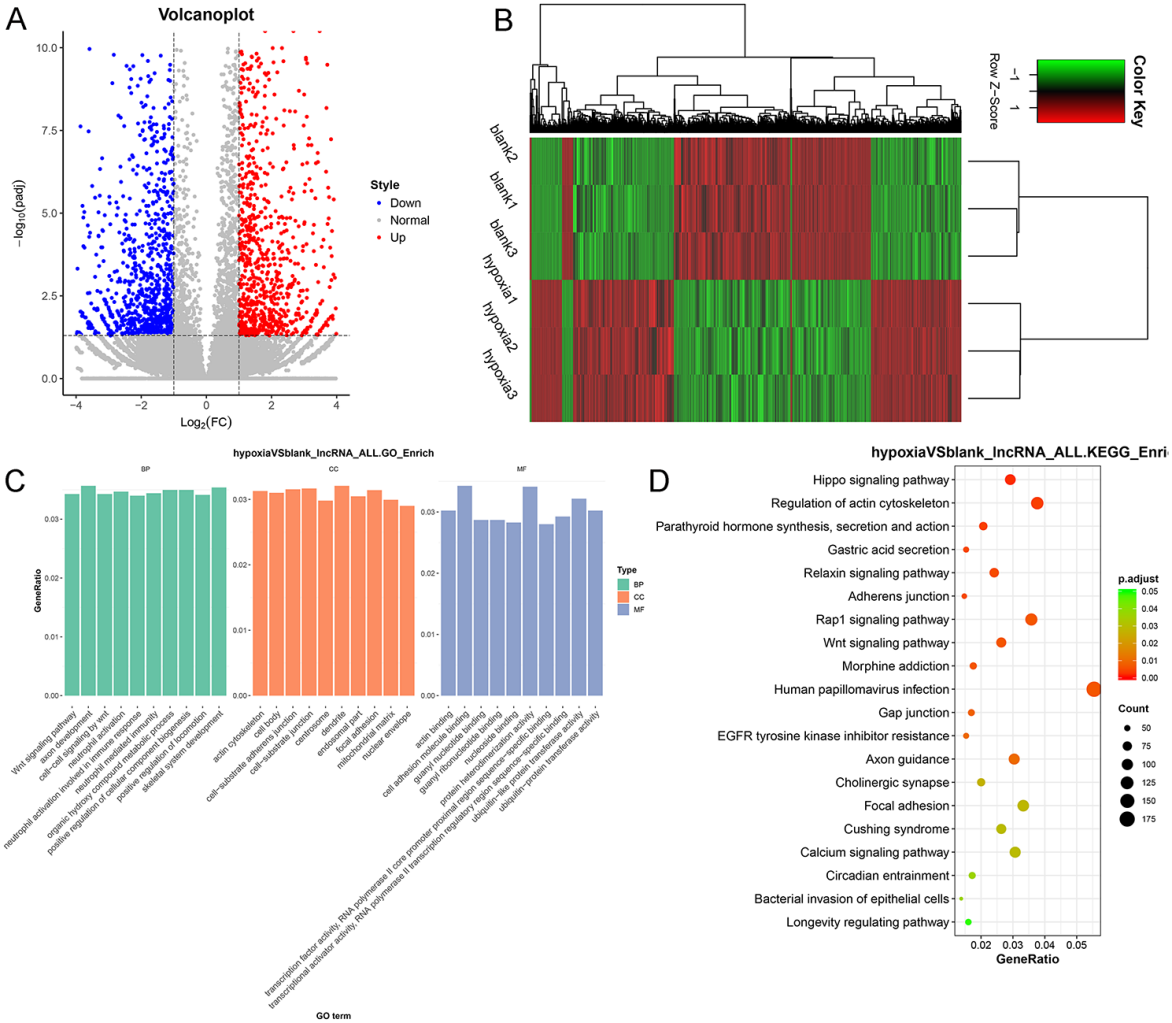
Identification of differential expressed long non-coding RNAs (DElncRNAs) and their functional analyses. (**A**) Volcano plots of DElncRNAs based on the thresholds of |log_2_Fold change (FC)| > 1 and adjusted P value (padj) < 0.05. Red and blue dots respectively represented the up-regulated and down-regulated DElncRNAs. (**B**) A bidirectional hierarchical clustering heatmap of the identified DElncRNAs. (**C**) Top10 gene ontology (GO) terms of biological process (BP), cellular component (CC) and molecular function (MF) of these identified DElncRNAs. (**D**) Significantly enriched Kyoto Encyclopedia of Genes and Genomes (KEGG) pathways of the identified DElncRNAs.

To further explore the molecular mechanisms underlying the hypoxic conditions in A549 cells, RNA sequencing was performed on normal A549 cells and hypoxic A549 cells. Based on the thresholds of |log_2_ FC| > 1 and padj < 0.05, 2426 DElncRNAs were identified between normal A549 cells and hypoxic A549 cells, including 1256 upregulated and 1170 downregulated DElncRNAs (Fig. 1A). These DElncRNAs were then used for hierarchical cluster analysis and were found to significantly distinguish normal A549 cells from hypoxic A549 cells (Fig. 1B). Next, these DElncRNAs were sent for GO terms, including biological process (BP), cellular component (CC), and molecular function (MF), and KEGG pathway enrichment analysis. The top10 GO terms for BP, CC, and MF were displayed in Fig. 1C. It was obvious that these identified DElncRNAs were significantly enriched in BP of “Wnt signaling pathway,” “axon development” and “neutrophil activation involved in immune response”; and in CC of “actin cytoskeleton,” “cell-substrate adherens junction,” and “mitochondrial matrix”; as well as in MF of “actin binding,” “cell adhesion molecule binding,” and “guanyl nucleotide binding.” KEGG pathway enrichment analysis showed that these DElncRNAs were significantly enriched in 24 KEGG pathways (padj < 0.05). The top20 KEGG pathways were shown Fig. 1D, including the “Hippo signaling pathway,” “relaxin signaling pathway,” “Rap1 signaling pathway,” “Wnt signaling pathway,” “EGFR tyrosine kinase inhibitor resistance,” “calcium signaling pathway” and “longevity regulating pathway”.

### Identification of DEmiRNAs between normal and hypoxic A549 cells and functional analysis of these DEmiRNAs

**Fig. 2:**
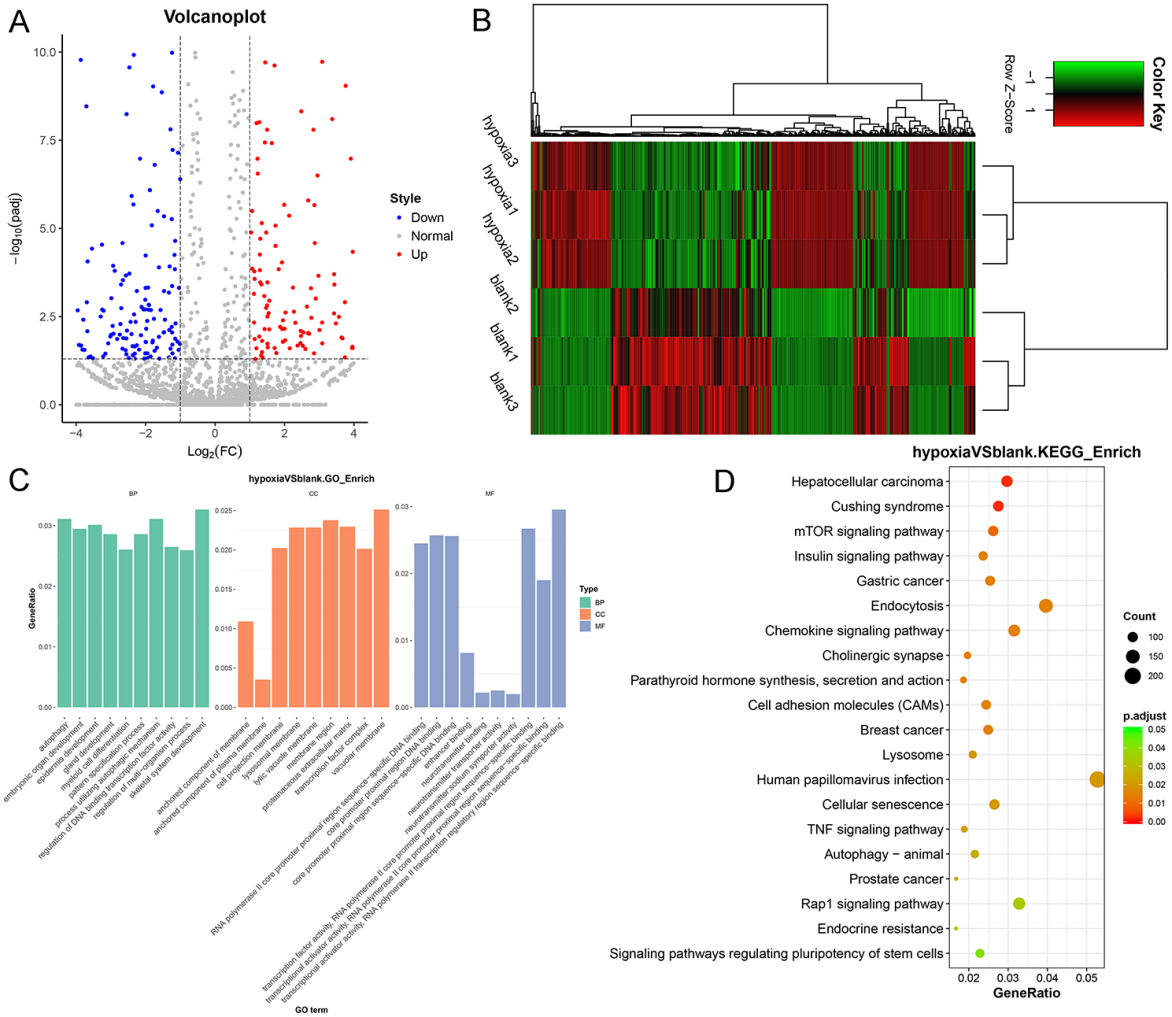
Analysis of differential expressed microRNAs (DEmiRNAs). (**A**) Volcano plots of DEmiRNAs based on the thresholds of |log_2_FC| > 1 and padj < 0.05. Red and blue dots respectively denoted the up-regulated and down-regulated DEmiRNAs. (**B**) A bidirectional hierarchical clustering heatmap of the screened DEmiRNAs. (**C**) GO terms of these screened DEmiRNAs. (**D**) Significantly enriched KEGG pathways of the screened DEmiRNAs.

After small RNA sequencing, 2787 miRNAs were annotated, and with the standards of |log_2_ FC| > 1 and padj < 0.05, 501 DEmiRNAs, including 234 upregulated DEmiRNAs and 267 downregulated DEmiRNAs, were screened (Fig. 2A). A bidirectional hierarchical clustering heatmap was used to analyze the upregulated or downregulated miRNAs in all normal and hypoxic A549 cells, and it was found that these DE miRNAs could differentiate between normal A549 cells and hypoxic A549 cells (Fig. 2B). Additionally, these screened DEmiRNAs were subjected to GO term and KEGG pathway analyses. Based on padj < 0.05, 78 GO terms and 29 KEGG pathways were significantly enriched. GO terms analysis showed that the significant enrichment terms of DEmiRNAs were related to BP of “skeletal system development,” “autophagy,” and “myeloid cell differentiation”; and CC of “vacuolar membrane,” “membrane region,” and “proteinaceous extracellular matrix”; and MF of “core promotor proximal region DNA binding,” “transcriptional activator activity, RNA polymerase II transcription regulatory region sequence-specific binding,” and “neurotransmitter transporter activity” (Fig. 2C). The significantly enriched KEGG pathways of the screened DEmiRNAs contained “mTOR signaling pathway”, “insulin signaling pathway”, “endocytosis”, “chemokine signaling pathway”, “TNF signaling pathway”, “autophagy-animal”, “Rap1 signaling pathway”, and “signaling pathways regulating pluripotency of stem cells” (Fig. 2D).

### Analysis of ceRNA networks

**Fig. 3:**
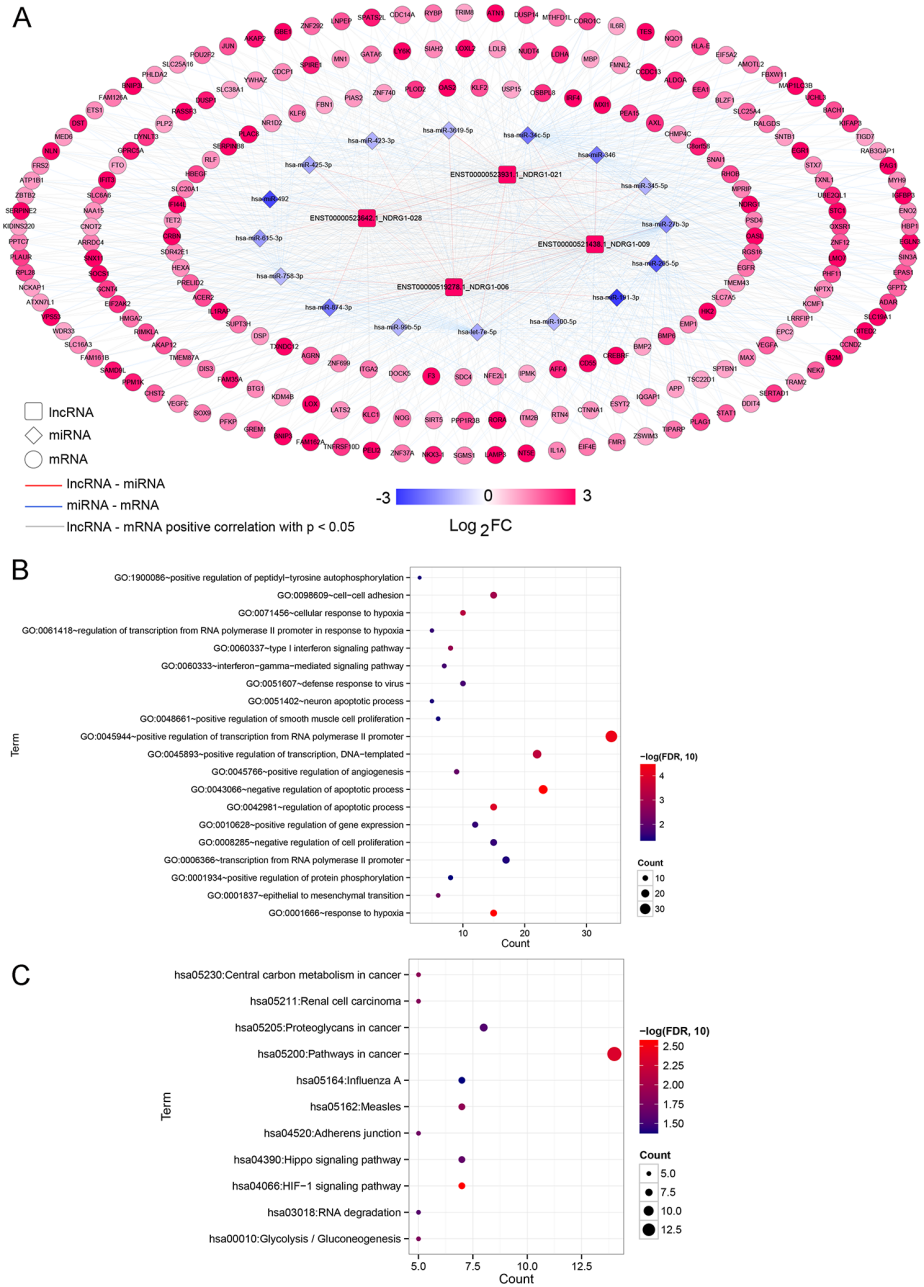
Construction of competitive endogenous RNA (ceRNA) neworks and functional analyses of these genes in the ceRNA networks. (**A**) CeRNA networks about lncRNA NDRG1. Squares, rhombuses, and circles represented lncRNAs, miRNAs, and mRNAs, respectively. (**B**) GO terms analysis of the genes in the ceRNA networks. (**C**) Significantly enriched KEGG pathway of the genes in the ceRNA networks

Due to the close connection between lncRNA NDRG1 and hypoxia, lncRNA NDRG1 was selected as the subject of this study. After filtering, ceRNA networks consisting of four lncRNA NDRG1 transcripts (NDRG1-006, NDRG1-009, NDRG1-028 and NDRG1-021), 16 miRNAs (hsa-miR-346, hsa-miR-34c-5p, hsa-miR-345-5p, hsa-miR-492, hsa-miR-191-3p, hsa-miR-205-5p, etc.) and 221 target mRNAs (VEGFA, ERG1, AKAP2, HK2, DUSP1, SERPINB8, etc.) were built (Fig. 3A). Subsequently, genes in the established ceRNA network were used for functional analyses. GO terms analysis showed that these genes in the ceRNA networks were associated with “cellular response to hypoxia”, “regulation of transcription from RNA polymerase II promoter in response to hypoxia”, “interferon-gamma-mediated signaling pathway”, “response to hypoxia”, “positive regulation of transcription from RNA polymerase II promotor”, “negative regulation of apoptotic process”, “positive expression of gene expresssion” and “type I interferon signaling pathway” (Fig. 3B). These genes in the ceRNA networks were also significantly involved in some KEGG pathways, including “pathways in cancer,” “Hippo signaling pathway,” “HIF-1 signaling pathway,” “RNA degradation,” and “glycolysis/gluconeogenesis” (Fig. 3C).

### Validation of ceRNA networks by RT-qPCR

**Fig. 4:**
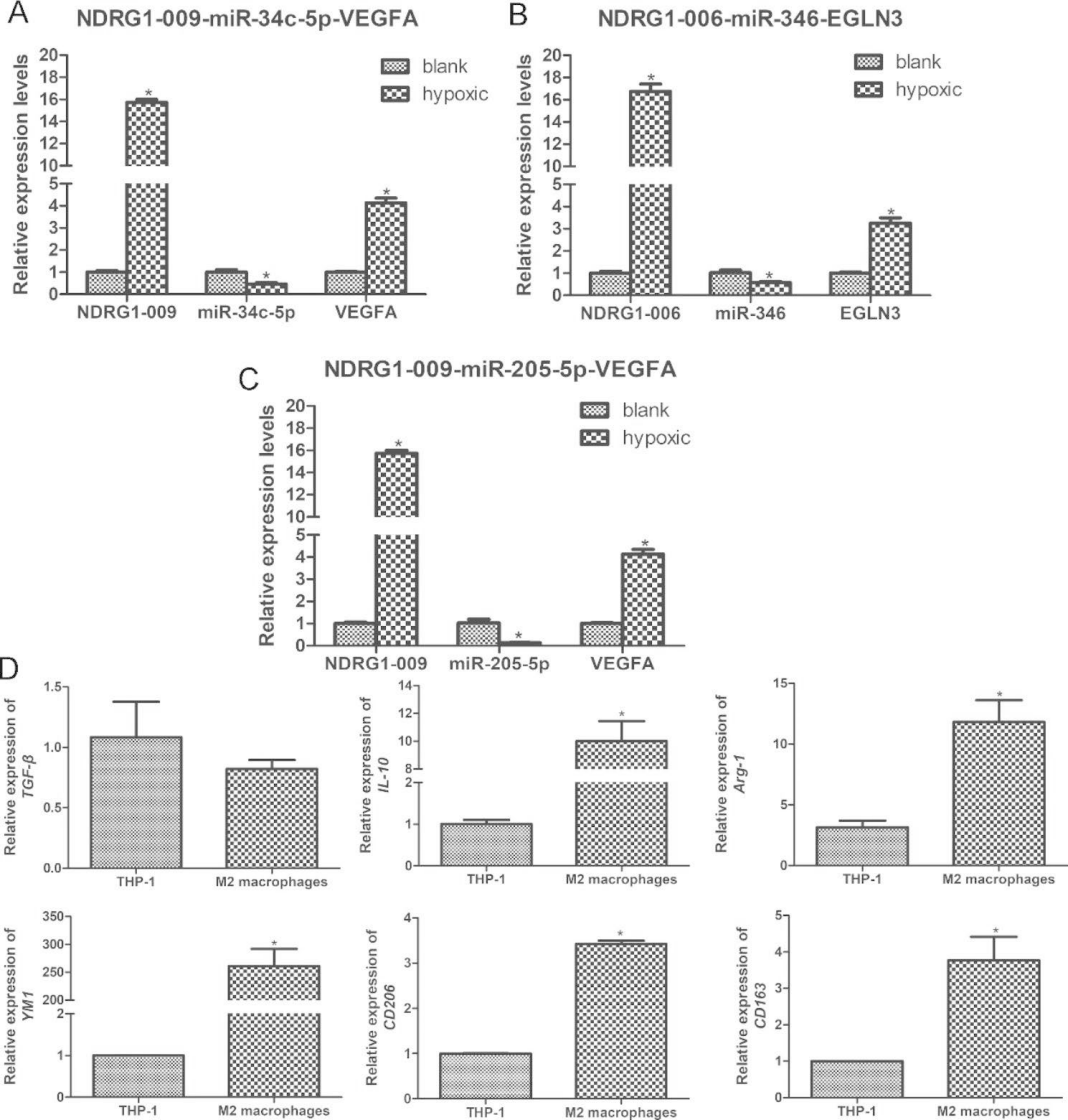
Validation of selected three ceRNA pairs and induction of M2 macrophages using real-time quantitative PCR. (**A**) The expression levels of lncRNA NDRG1-009-miR-34c-5p-VEGFA. (**B**) The expression levels of lncRNA NDRG1-006-miR-346-EGLN3. (**C**) The expression levels of lncRNA NDRG1-009-miR-205-5p-VEGFA. *: *P* < 0.05, compared with the blank group. (**D**) The relative expression of M2 macrophages-related markers TNF-β, IL-10, Arg-1, YM1, CD206, and CD163 in the THP-1 cells and M2 macrophages. *: *P* < 0.05, compared with the THP-1 cells

To verify the reliability of the sequencing, three ceRNA pairs (NDRG1-009-miR-34c-5p-VEGFA, NDRG1-006-miR-346-EGLN3, and NDRG1-009-miR-205-5p-VEGFA) were selected for RT-qPCR validation. It is clear that the expression of NDRG1-009 and NDRG1-006 was both significantly upregulated in hypoxic A549 cells compared to normal A549 cells (*P* < 0.05), while the levels of miR-34c-5p, miR-346, and miR-205-5p were all significantly decreased in hypoxic A549 cells (*P* < 0.05, Fig. 4A-C). The expression of VEGFA and EGLN3 in the blank and hypoxic groups was similar to that of lncRNA NDRG1-009 (Fig. 4A-C). These results showed that the expression patterns of the genes in these three ceRNA pairs were consistent with the gene expression trends in the ceRNA networks and sequencing results, indicating a high relative reliability of the sequencing outcomes.

### Characterization of EVs and cellular uptake of the EVs

THP-1 cells were used to induce M2 macrophages through treated with PMA, IL-4, and IL-13, and the M2 macrophages-markers (TGF-β, IL-10, Arg-1, YM1, CD206, and CD163) were measured to evaluate the formation of M2 macrophages. There was no significant difference in TGF-β expression between THP-1 cells and induced cells (*P* > 0.05, Fig. 4D). The expression levels of IL-10, Arg-1, YM1, CD206, and CD163 were significantly higher in M2 macrophages than in THP-1 cells (*P* < 0.05; Fig. 4D), implying that M2 macrophages were successfully induced by PMA, IL-4, and IL-13.

**Fig. 5:**
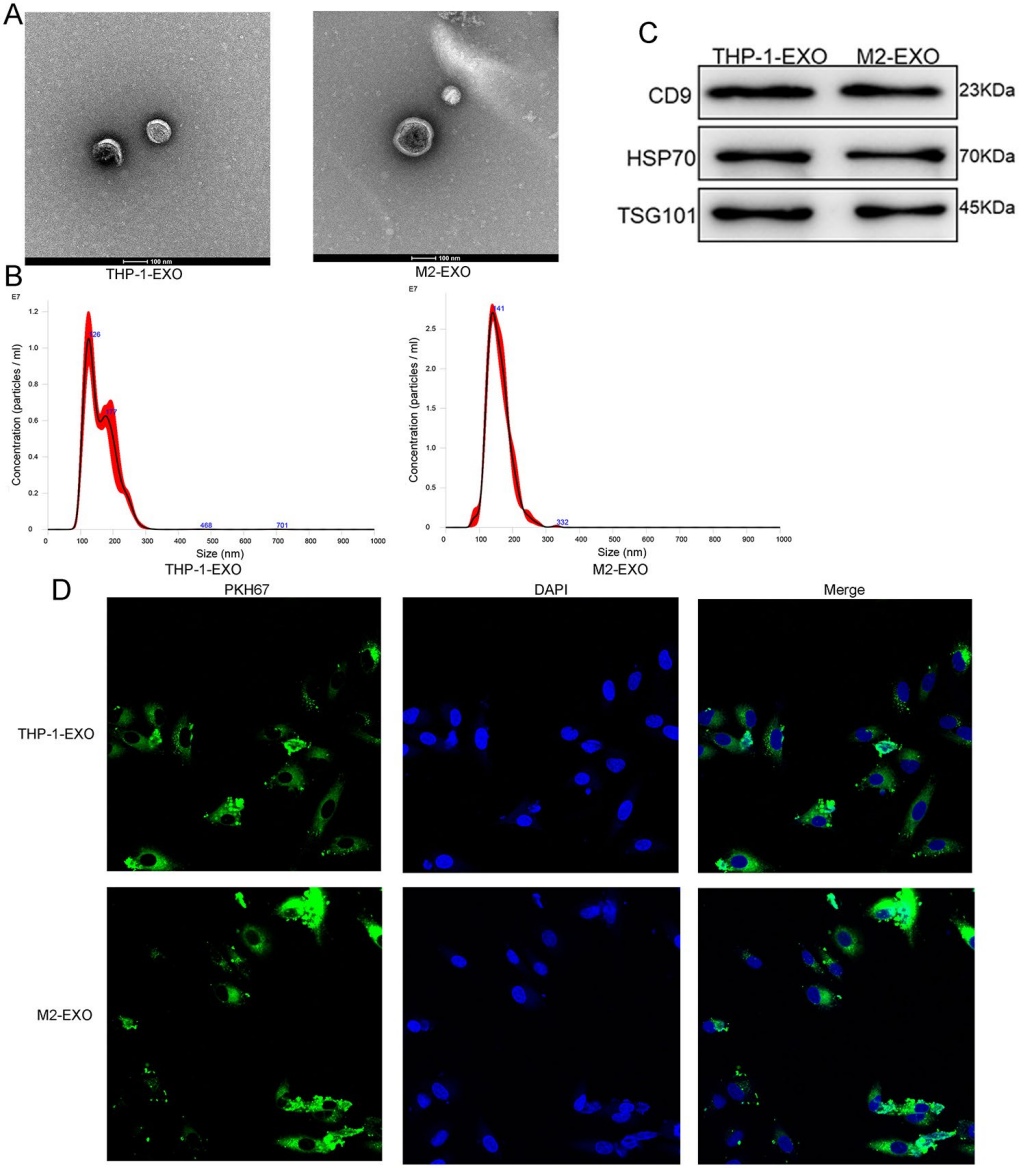
Characterization of the extracellular vesicles (EVs) from THP-1 cells and M2 macrophages. (**A**) The morphology of EVs isolated from THP-1 cells and M2 macrophages was observed by a transmission electron microscopy. (**B**) A Nanosight NS300 particle size analyser was used to measure the particle size of THP-1 cells-derived EVs and M2 macrophages-derived EVs. (**C**) Western blot was used to detect the expression of the EVs-specific markers (CD9, HSP70 and TSG101). (**D**) Cellular uptake of PKH67-labeled exosomes (green fluorescence) and hypoxic A549 cells after co-cultured for 48 h. hy-A549: hypoxic A549 cells; THP-1-EVs: THP-1 cells-derived EVs; M2-EEVs: M2 macrophages-derived EVs.

Successfully induced M2 macrophages and THP-1 cells were collected for EVs isolation, and TEM, NTA, and western blotting were used to characterize the EVs. The TEM results showed that the substances isolated from THP-1 cells and M2 macrophages both exhibited a cup shape or were nearly round with a diameter of approximately 100 nm (Fig. 5A, **Figure S2**). NTA revealed that the major peaks of the substances derived from THP-1 cells and M2 macrophages were 126 and 141 nm, respectively (Fig. 5B), and the particle size distribution was approximately 100–200 nm, which was in line with the size distribution of EVs, as previously reported [32]. Furthermore, western blotting analysis showed that the EV-specific markers CD9, HSP70 and TSG101, which are the EVs specific markers, were expressed in THP-1 cells-derived EVs and M2 macrophage-derived EVs (Fig. 5C, **Figure S3**). These results demonstrate that EVs were successfully extracted from THP-1 cells and M2 macrophages.

To observe the uptake of THP-1 cell-derived EVs and M2 macrophage-derived EVs by hypoxic A549 cells, PKH67 was used to label EVs with green fluorescence, and DAPI was used to stain hypoxic A549 cells with blue fluorescence. After co-culture for 48 h, most cells showed intracellular fluorescence (Fig. 5D), which indicated that THP-1 cell-derived EVs and M2 macrophage-derived EVs were taken up by the hypoxic A549 cells.

### Effects of M2 macrophages-derived EVs on the growth of hypoxic A549 cells

**Fig. 6:**
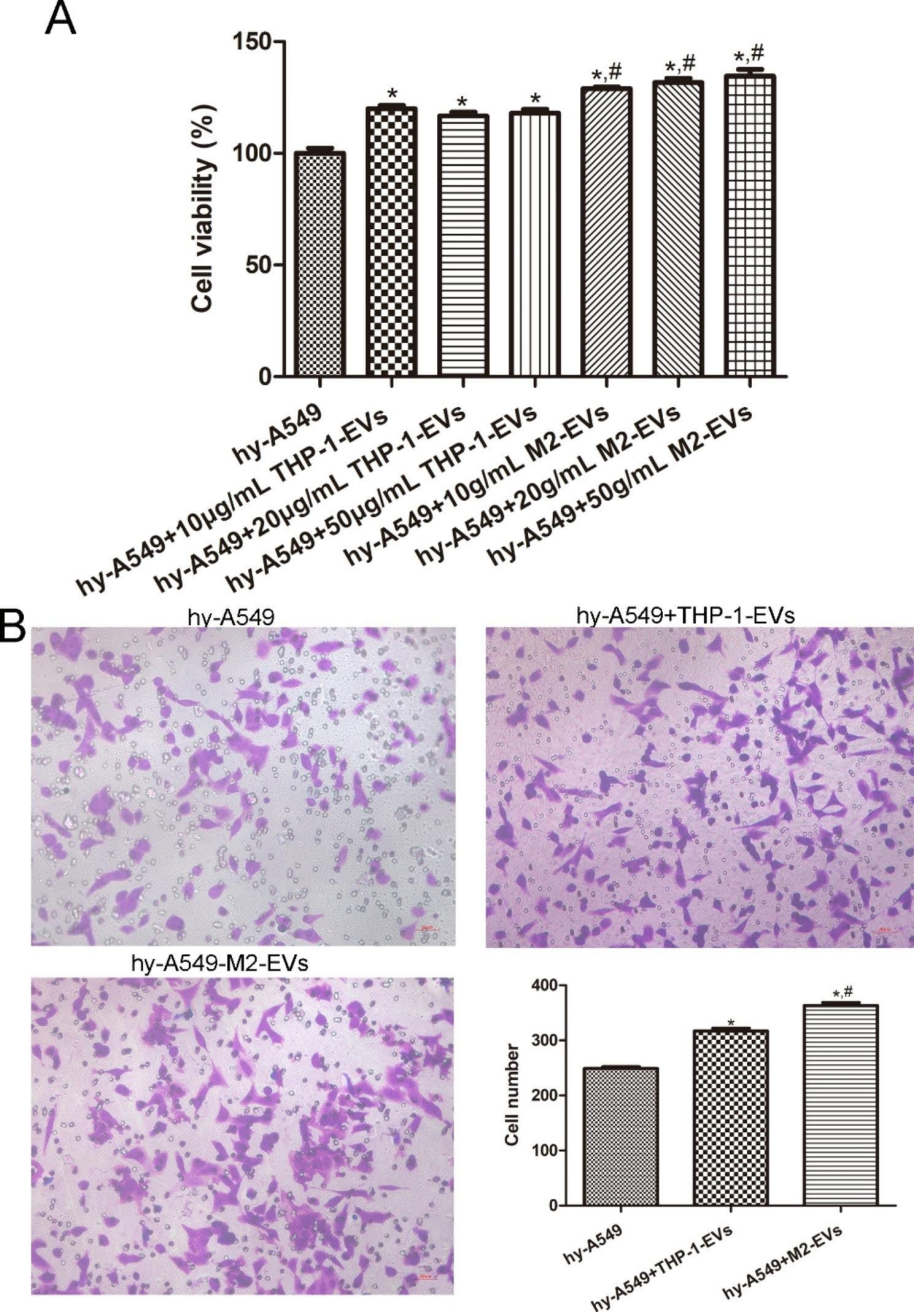
Effects of M2 macrophages-derived EVs on the growth of hypoxic A549 cells. (**A**) Cell viability of hypoxic A549 cells treated with different concentrations of different EVs was determined by cell counting kit-8. (**B**) Cell migration of hypoxic A549 cells treated with different EVs was measured using Transwell. *: *P* < 0.05, compared with the hypoxic A549 cells. ^#^: *P* < 0.05, compared with the hypoxic A549 cells treated with THP-1 cells-derived EVs. hy-A549: hypoxic A549 cells; THP-1-EVs: THP-1 cells-derived EVs; M2-EVs: M2 macrophages-derived EVs.

To further investigate the role of M2 macrophage-derived EVs in hypoxic A549 cells, different EVs were used to treat hypoxic A549 cells, and CCK-8 and Transwell assays were used to determine cell viability and migration. No significant differences were found in cell viability among the hypoxic A549 cells treated with 10 µg/mL, 20 µg/mL, and 50 µg/mL THP-1 cells-derived EVs; as well as among the hypoxic A549 cells treated with 10 µg/mL, 20 µg/mL, and 50 µg/mL M2 macrophages-derived EVs (*P* > 0.05, Fig. 6A). Therefore, 10 µg/mL THP-1 cells-derived EVs and M2 macrophages-derived EVs were chosen for following experiments. Moreover, compared to hypoxic A549 cells, the viability of cells treated with THP-1 cell-derived EVs and M2 macrophage-derived EVs was significantly enhanced (*P* < 0.05), and the effects of M2 macrophage-derived EVs were more significant than those of THP-1 cell-derived EVs (*P* < 0.05, Fig. 6A).

Transwell results showed that the cell number in the hypoxic A549 cells, THP-1 cells-derived EVs-treated hypoxic A549 cells and M2 macrophages-derived EVs-induced hypoxic A549 cells was 248.8 ± 7.19, 316.8 ± 11.21, and 363.2 ± 11.52, respectively (Fig. 6B). The results illustrated that THP-1 cells-derived EVs and M2 macrophages-derived EVs significantly promoted the migration of hypoxic A549 cells (*P* < 0.05), and that the action of M2 macrophage-derived EVs was better than that of THP-1 cell-derived EVs (*P* < 0.05, Fig. 6B).

### Effects of M2 macrophages-derived EVs on the expression of the related RNAs in the hypoxic A549 cells

**Fig. 7:**
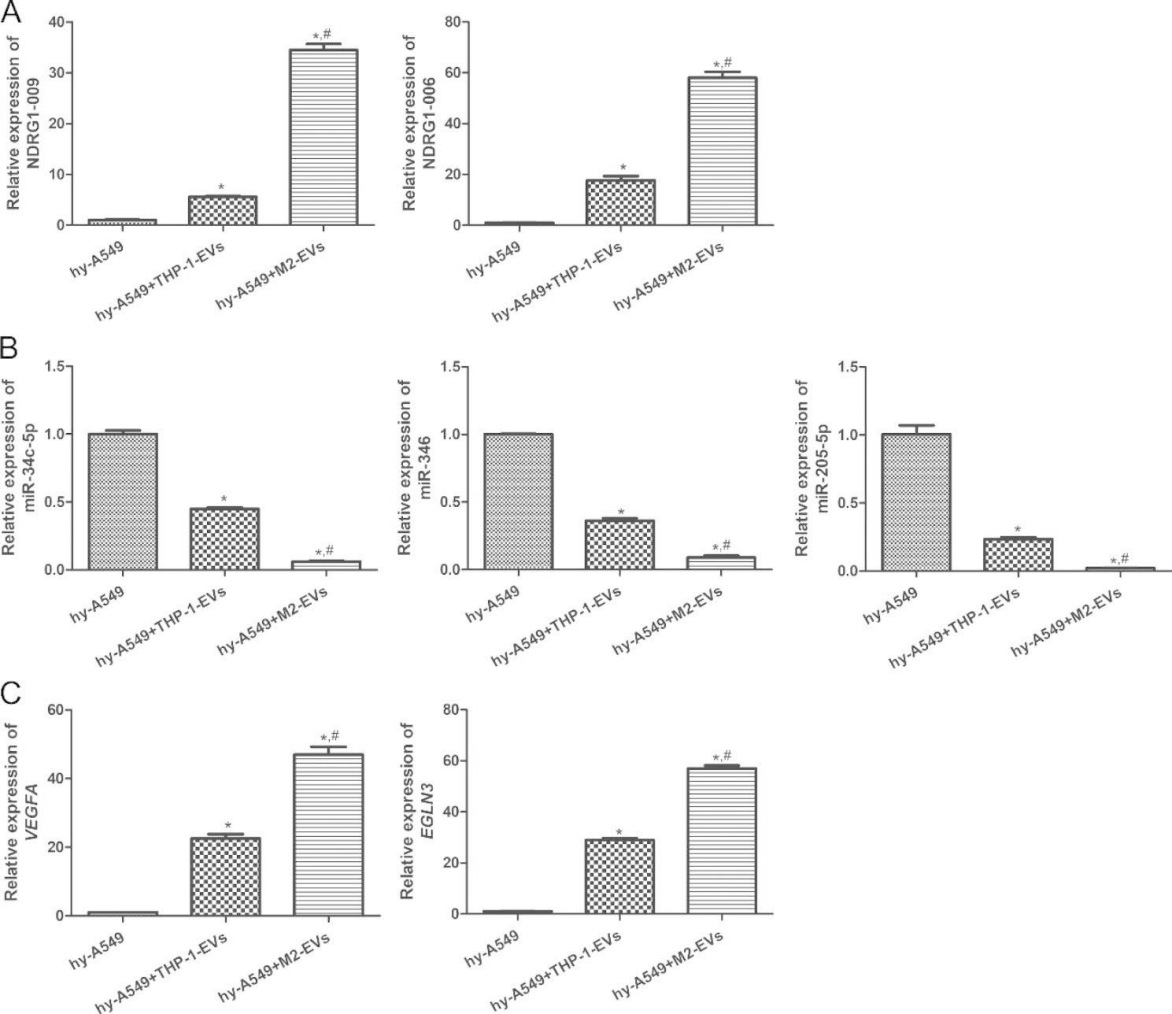
Effects of M2 macrophages-derived EVs on the expression of the related lncRNAs, miRNAs and mRNAs in the hypoxic A549 cells by RT-qPCR. (**A**) The mRNA expression of lncRNA NDRG1-009 and NDRG1-006. (**B**) The mRNA expression of miR-34c-5p, miR-346 and miR-205-5p. (**C**) The mRNA expression of *VEGFA* and *EGLN3*. *: *P* < 0.05, compared with the hypoxic A549 cells. ^#^: *P* < 0.05, compared with the hypoxic A549 cells treated with THP-1 cells-derived EVs. hy-A549: hypoxic A549 cells; THP-1-EVs: THP-1 cells-derived EVs; M2-EVs: M2 macrophages-derived EVs.

After EVs treatment, the expression of the lncRNAs NDRG1-009 and NDRG1-006, miRNAs miR-34c-5p, miR-346, and miR-205-5p, and mRNA *VEGFA* and *EGLN3*. The expression of lncRNAs NDRG1-009 and NDRG1-006 was up-regulated in the EV-treated hypoxic A549 cells compared with the hypoxic A549 cells (*P* < 0.05), and their expression in the hy-A549 + M2-EVs group was significantly higher than that in the hy-A549 + THP-1-EVs group (*P* < 0.05, Fig. 7A). For miR-34c-5p, miR-346, and miR-205-5p, levels in the EV-treated groups were evidently lower than those in the hy-A549 cells (*P* < 0.05), and M2 macrophage-derived EVs had a more significant effect on their levels than THP-1 cell-derived EVs (*P* < 0.05, Fig. 7B). Additionally, the trend of *VEGFA* and *EGLN3* mRNA expression in the different groups was opposite to that of miRNA levels (Fig. 7C). These results implied that M2 macrophage-derived EVs could further upregulate the expression of NDRG1-009, NDRG1-006, *VEGFA* and *EGLN3* while downregulating miR-34c-5p, miR-346, and miR-205-5p in hypoxic A549 cells.

## Discussion

NSCLC is the leading cause of cancer-related death worldwide and is a serious threat to people’s life and health. Hypoxia contributes to the development of invasive and metastatic cancer cells, and is detrimental to cancer treatment [33]. Our study found that hypoxic conditions enhanced the viability of A54 cells, implying that a hypoxic environment may accelerate the growth of A549 cells. Therefore, RNA sequencing was used to further to explore the molecular mechanisms underlying the hypoxic microenvironment in NSCLC development. After analysis, 2426 DElncRNAs (1256 up-regulated and 1170 down-regulated) and 501 DEmiRNAs (234 upregulated and 267 downregulated) were identified between normal A549 cells and hypoxic A549 cells. These DElncRNAs and DEmiRNAs were significantly enriched in “Wnt signaling pathway,” “Hippo signaling pathway,” “Rap1 signaling pathway,” “calcium signaling pathway,” “mTOR signaling pathway,” and “TNF signaling pathway.”

The Wnt signaling pathway is closely related to tissue development and homeostasis by regulating endogenous stem cells in tissues, and abnormal Wnt signaling has been described as a key player in the genesis, maintenance, and development of many cancers by influencing the behavior of cancer stem cells [34]. Yang et al. [35] reported that FOXP3 acted as a coactivator to promote Wnt/β-catenin signaling pathway, and induce EMT, growth and metastasis of NSCLC cells. The Hippo signaling pathway is an evolutionarily conserved pathway that plays key roles in organ development, epithelial homeostasis, tissue regeneration, wound healing, and immune regulation [36]. Wang et al. demonstrated that PDK-1 knockdown could induce apoptosis in NSCLS cells by blocking the expression of YAP and IRS2, dependent on the Hippo-YAP signaling pathway, which is involved in NSCLC occurrence and progression [37]. There is considerable evidence that the Rap1 signaling pathway is critical for a range of cell functions, including cell proliferation, differentiation, and adhesion [38, 39]. Moreover, some key Rap1 functions depend on crosstalk with the calcium signaling pathway and are associated with cancer initiation and development [38]. A previous report showed that GREM1 overexpression could promote the migration, invasion, and EMT of A549 cells by upregulating Rap1 pathway intermediaries, thereby contributing to NSCLC progression [40]. TNF, an inflammatory cytokine, can activate the NF-κB signaling pathway by binding to its receptor TNF-R1 and plays an important role in cancer development and progression [41]. In addition, the mTOR signaling pathway has been found to be upregulated in HCC tissues compared to the surrounding cirrhotic tissues and is involved in many cancer markers, including cell growth, metabolic reprogramming, proliferation, and inhibition of apoptosis [42]. Zhang et al. [43] found that up-regulation of pro-inflammatory cytokines (IL-1β, IL-6 and TNF-α) in periaqueductal gray matter of cancer rats amplified PI3K-mTOR signal in this brain region and changed the descending pathway of pain transmission; thus, facilitating the occurrence of bone cancer-induced pain. Based on our results, we hypothesized that hypoxia may aggravate the occurrence and development of NSCLC by mediating the Wnt, Hippo, Rap1, calcium, mTOR, and TNF signaling pathways.

LncRNA-associated ceRNA networks can reveal the underlying molecular mechanisms of various diseases, thus unearthing new biomarkers and therapeutic targets related to these diseases [44]. Based on the identified DElncRNAs, we found that lncRNA NDRG1 expression was significantly different between normal A549 cells and hypoxic A549 cells. After searching the literature, it has been reported that NDRG1-OT1, an oxygen-responsive lncRNA in breast cancer cells, was differentially expressed in hypoxic breast cancer cells [45]. Therefore, our research constructed ceRNA networks composed of four lncRNA NDRG1 transcripts, 16 miRNAs and 221 target mRNAs, and functional analyses showed that these genes in the ceRNA networks were significantly enriched in “Hippo signaling pathway” and “HIF-1 signaling pathway.” RT-qPCR confirmed that the expression patterns of three ceRNA pairs (NDRG1-009-miR-34c-5p-VEGFA, NDRG1-006-miR-346-EGLN3, and NDRG1-009-miR-205-5p-VEGFA) were consistent with the sequencing data. Ye et al. [46] clarified that VEGFA could bind with miR-34c-5p, and its expression was positively correlated with LINC01123 expression; in addition, LINC01123 had been shown to modulate colon cancer progression and cytochemical resistance through miR-34c-5p/VEGFA axis. MiR-205-5p is involved in VEGF-associated angiogenesis and appears to regulate cellular signaling pathways such as cell migration, proliferation, and apoptosis [47]. A previous study showed that miR-205-5p overexpression suppressed the growth and EMT of renal cell carcinoma (RCC) cells by targeting the VEGFA and PI3K/Akt signaling pathways, thus serving as a tumor suppressor in RCC [48]. MiR-346 has also been found to be associated with the development of cancers such as HCC [49], NSCLC [50], and gastric cancer (GC) [51]. EGLN3, a key gene that adapts to the hypoxic microenvironment, has various biological functions such as regulation of cell signaling, metabolism, cell cycle, and migration of cancer cells, and plays a vital role in tumor growth and progression [52]. In addition, increased expression of HIF-1α is a key feature of hypoxic environment, which mediates the transcription of genes related to oxygen transfer and hypoxia metabolic adaptation, and plays an essential role in tumor proliferation, metabolism, angiogenesis, metastasis, and differentiation [53]. These reports combined with our results suggest that a hypoxic microenvironment may worsen the progression of NSCLC by regulating the NDRG1-009-miR-34c-5p-VEGFA, NDRG1-006-miR-346-EGLN3, NDRG1-009-miR-205-5p-VEGFA, and Hippo/HIF-1 signaling pathways.

M2 macrophages can be polarized by various stimulators, including cytokines (IL-4, IL-10, and IL-13), glucocorticoids, immune complexes, and lipopolysaccharide [54]. M2 macrophages can clear apoptotic cells, reduce inflammatory responses, and promote wound healing. However, they can also cause allergic inflammation, contribute to tumor tissue growth, and act as cellular reservoirs for various pathogens [55]. A study of Lei et al. illustrated that M2 macrophages-derived EVs transferred miR-501-3p to lung cancer cells could promote the growth of lung cancer cells through down-regulating WDR82 expression, thereby intensify lung cancer progression [56]. Another study showed that EVs isolated from M2 macrophages could transmit miR588 into GC cells and enhance their resistance of GC cells to cisplatinum by targeting CYLD [57]. These results indicated the importance of M2 macrophage-derived EVs in multiple cancers. Our study shows that EVs derived from M2 macrophages-derived EVs significantly enhance the viability and migration of hypoxic A549 cells. Taken together, we speculate that M2 macrophage-derived EVs may promote NSCLC development in a hypoxic microenvironment.

In addition, we found that M2 macrophage-derived EVs further upregulated the expression of NDRG1-009, NDRG1-006, *VEGFA* and *EGLN3* while downregulating miR-34c-5p, miR-346, and miR-205-5p in hypoxic A549 cells. M2-polarized macrophages in the tumor microenvironment promote cell growth through EVs transfer. A previous study showed that miR-186-5p was abundant in M2 macrophage-derived EVs, and M2 macrophages-derived EVs delivered miR-186-5p to cells by targeting DLC1 expression, thereby enhancing the growth and motility of colon cancer cells by activating the β-catenin pathway [58]. Another study found that M2 macrophages-derived EVs carried miR-21-5p entered into CD8 T cells to inhibit YOD1 expression and activate YAP/β-catenin pathway, thus promoting CD8 T cell depletion in hepatocellular carcinoma, which provided new insights into hepatocellular carcinoma immunotherapy [59]. In summary, M2 macrophage-derived EVs may aggravate NSCLC under hypoxic conditions via three identified axes (NDRG1-009-miR-34c-5p-VEGFA, NDRG1-006-miR-346-EGLN3, and NDRG1-009-miR-205-5p-VEGFA).

It should be noted that this study has some limitations. First, further experiments (RNA interference or overexpression methods) are needed to investigate the specific molecular mechanisms of the identified ceRNA pairs in NSCLC progression in a hypoxic microenvironment, and the specific effects of the Wnt, Hippo, Rap1, calcium, mTOR, and TNF signaling pathways in NSCLC under hypoxic conditions should be further verified. In addition, whether M2 macrophage-derived EVs can inhibit or accelerate the growth of hypoxic NSCLC cells through the identified ceRNA pairs should be studied in the future.

## Conclusions

In conclusion, RNA sequencing identified 2426 DElncRNAs and 501 DEmiRNAs between normal and hypoxic A549 cells, and functional analyses showed that Wnt, Hippo, Rap1, calcium, mTOR, and TNF may play important roles in hypoxic NSCLC. Based on the ceRNA networks, we found that the hypoxic microenvironment may worsen the progression of NSCLC by regulating the NDRG1-009-miR-34c-5p-VEGFA, NDRG1-006-miR-346-EGLN3, NDRG1-009-miR-205-5p-VEGFA, and Hippo/HIF-1 signaling pathways. Additionally, M2 macrophage-derived EVs may aggravate NSCLC development in a hypoxic microenvironment via three axes. Our work reveals the ceRNA mechanisms of hypoxia in NSCLC progression and improves our understanding of NSCLC in a hypoxic microenvironment.

## Electronic supplementary material

Below is the link to the electronic supplementary material.

Supplementary Material 1

## Data Availability

The raw data of sequencing openly available in a public repository that issues datasets with the accession number of PRJNA899343 in the NCBI SRA database (https://dataview.ncbi.nlm.nih.gov/object/PRJNA899343?reviewer=llqae24m4irqit1mi2bvum1m6e). Additionally, other data generated or analysed during this study are available from the corresponding author on reasonable request.
