# Supplementary material for: Roles of hypoxic environment and M2 macrophage-derived extracellular vesicles on the progression of non-small cell lung cancer
Source: BMC Pulm Med. 2023 Jul 3;23:239. doi: 10.1186/s12890-023-02468-7 (PMC10318818; doi:10.1186/s12890-023-02468-7)
Supplement: Supplementary file 1 — Supplementary Material 1 [file 12890_2023_2468_MOESM1_ESM.docx]

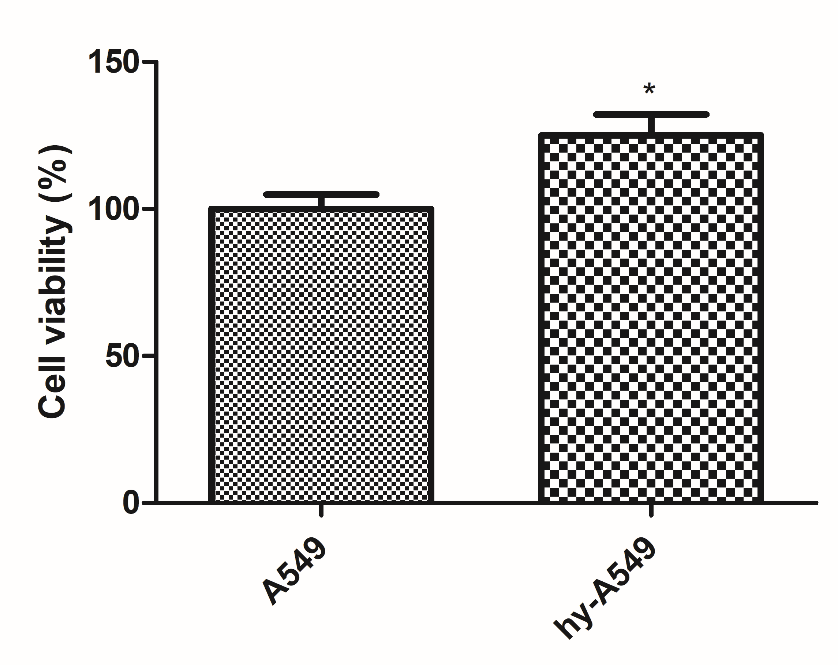


Figure S1 The viability of A549 cells under normal and hypoxic conditions using cell counting kit-8. *: *P* < 0.05, compared with the A549 cells. A549: the cells under normal conditions; hy-A549: the cells under hypoxic conditions.


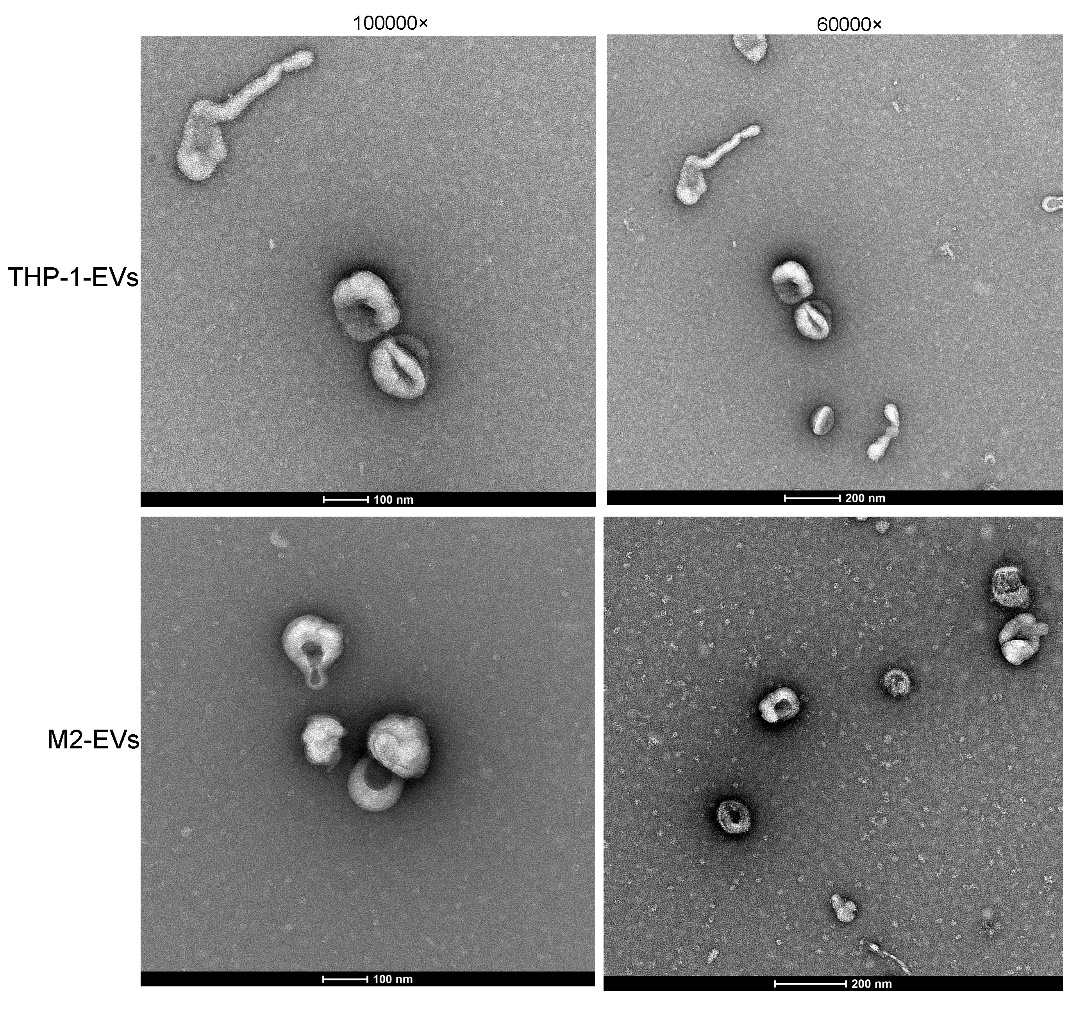


Figure S2 The transmission electron microscopy images of extracellular vesicles isolated from THP-1 cells and M2 macrophages at magnification of 60000× and 100000×.


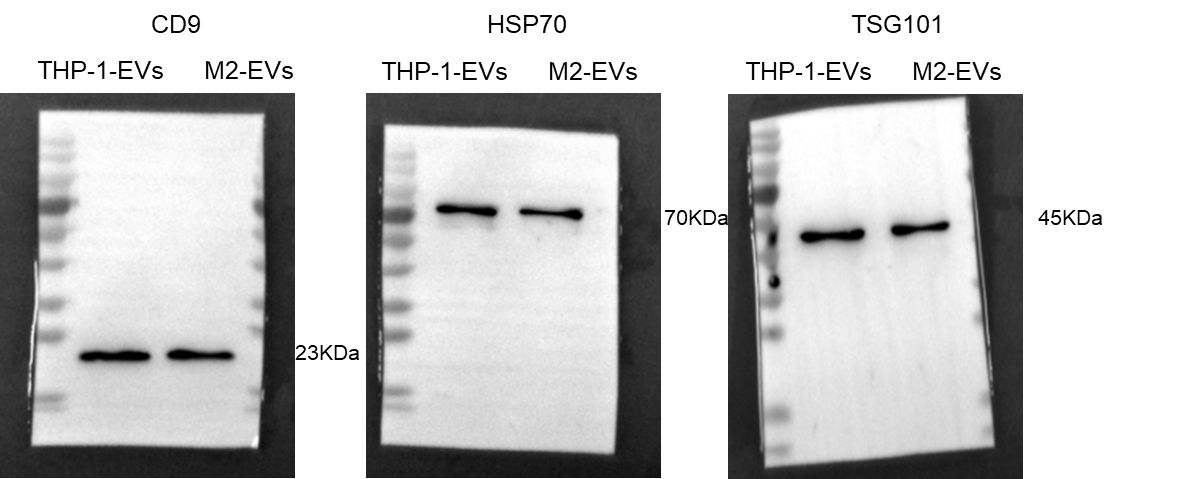


Figure S3 The original western blot images of CD9, HSP70 and TSG101 in the different extracellular vesicles.
